# Urology research publications: lessons learned from a developing country

**DOI:** 10.1186/1756-0500-7-429

**Published:** 2014-07-05

**Authors:** Fayez T Hammad

**Affiliations:** 1 Departments of Surgery, College of Medicine and Health Sciences, United Arab Emirates University, PO Box 17666, Al Ain, United Arab Emirates

**Keywords:** Urology, Research, Developing country

## Abstract

**Background:**

There is very limited data on the nature and type of published research in the field of Urology in the developing countries. Therefore, the aim of this study was to investigate the Urology research activities in one of the Gulf counties (United Arab Emirates (UAE) to define areas of deficiencies and challenges in a trial to learn some lessons to improve research in developing countries.

**Results:**

A total of 96 manuscripts were published from UAE up to 2012. The first publication was in 1985. There is an overall increase in the number of manuscripts published per year over time (one manuscript in 1985 vs. 10 manuscripts in 2012). Forty seven (49%) manuscripts came from the main academic institute (UAE University) and the rest came from the many hospitals around the country. There were 68 original research articles, 4 reviews and 24 case reports. 64 manuscripts represented clinical whereas 32 represented laboratory-based research. The manuscripts represented various urology subspecialties, the commonest of which was urinary obstruction and urolithiasis (n = 19). None of the manuscripts represented a proper epidemiological research. The research activities were sporadic and driven by individuals during particular periods with lack of continuity.

**Conclusions:**

Despite the increasing number of urology articles in this developing country, there is a lack of proper epidemiological studies to identify the local prevalence and behaviour of various conditions. There is also a lack of research groups and hence a lack of continuity of research in any particular field as the research is usually driven by specific research-oriented individuals the departure of who results in a cease in the research activities. Moreover, there is an evidence of lack of research training and capability in the majority of institutes. These observations might assist policy decision makers to develop research in this country and other developing countries.

## Background

Similar to other Gulf countries and probably other developing nations, United Arab Emirates (UAE) has observed a tremendous recent improvement in the medical services provided to its residents. Indeed, there has been a proliferation in the number of hospitals and physicians in the country. With the development of these services, it is expected to find a parallel improvement in the research activities in different medical subspecialties. Obviously, medical research is essential to study the local epidemiology and impact of various diseases in the national set up and to help in planning future strategies. In addition, it improves the quality of the medical services by encouraging evidence-based medical practice and by improving the critical thinking ability of the physicians as research-oriented individuals are more capable of critically appraising the published literature. Moreover, research activities, improves the standards in post-graduate training and might be a requirement for promotion in some institutes.

The overall number of medical publications from the UAE has been shown to increase overtime [1]. However, there have been limited reports on the nature and quality of publications in specific medical specialties [2]. Obviously, analyzing the quality, trends and defects in the research publications in a particular subspecialty is more relevant to healthcare planners because this provides them with the specific data required for future planning in a particular field. In urology, this data is not available. Therefore, the aim of this study was to investigate the nature and trends in the urology research publications in the UAE in a trial to learn some lessons to improve research in developing countries.

## Methods

A Midline (PubMed) [3] search of all urology-related manuscripts which originated from the UAE up to December 2012 was performed. The search used a combination of words related to both research origin (region) and research field. The words used to indicate research origin were: United Arab Emirates, UAE, Emirates, Gulf, Abu Dhabi, Dubai, Sharjah, Ajman, Umm Al-Quwain, Ras Al Khaimah and Fujairah whereas the words used to indicate the research field were: urology, urinary, uro, kidney, ureter, bladder, prostate, seminal vesicles, urethra, penis, scrotum and testis. All the manuscripts obtained under these headings were reviewed and only urology-related articles originated from UAE and written in English were included. Data related to the year of publication, address and type of article, field of research, whether the article represented clinical or laboratory-based research and the impact factor of the journal, were extracted. The impact factor considered in this study was the past 5 year impact factor as listed in the Journal Citation Reports in the ISI Web of knowledge website [4].

## Results

**Figure 1:**
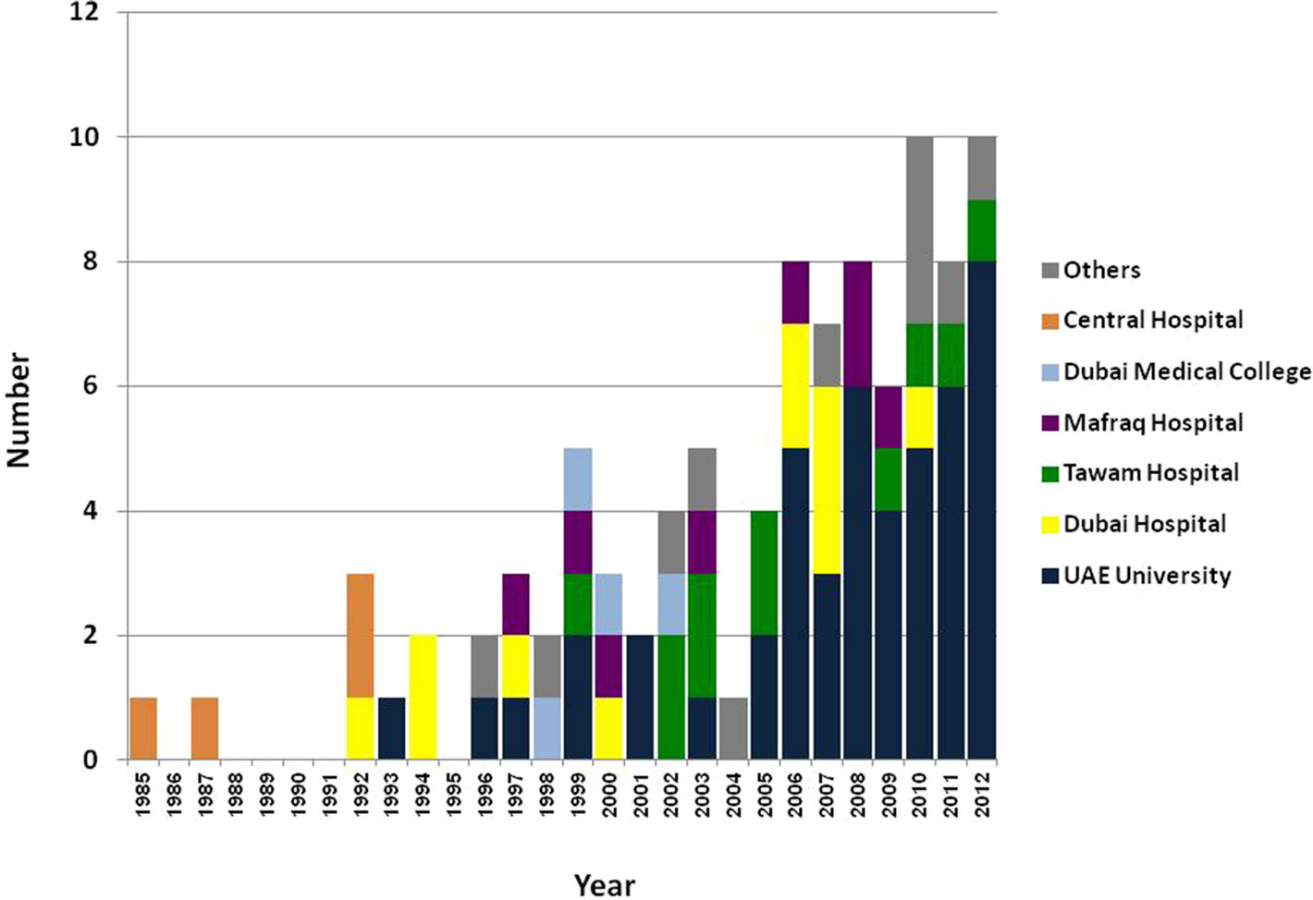
Number of urology publications per year per institute up to 2102.

A total of 96 manuscripts from UAE were published in indexed peer-reviewed journals up to December 2012. The first publication was in 1985. As demonstrated in Figure 1, there was an overall increase in the number of manuscripts published per year over time (one manuscript in 1985 vs. 10 manuscripts in 2012). The maximum number of annual publications was 10 articles per year and occurred in 2010 and 2012. Forty seven publications (49%) came from the main academic institute (UAE University) and the rest came from the many hospitals and institutes around the country (Figure 1).

**Figure 2:**
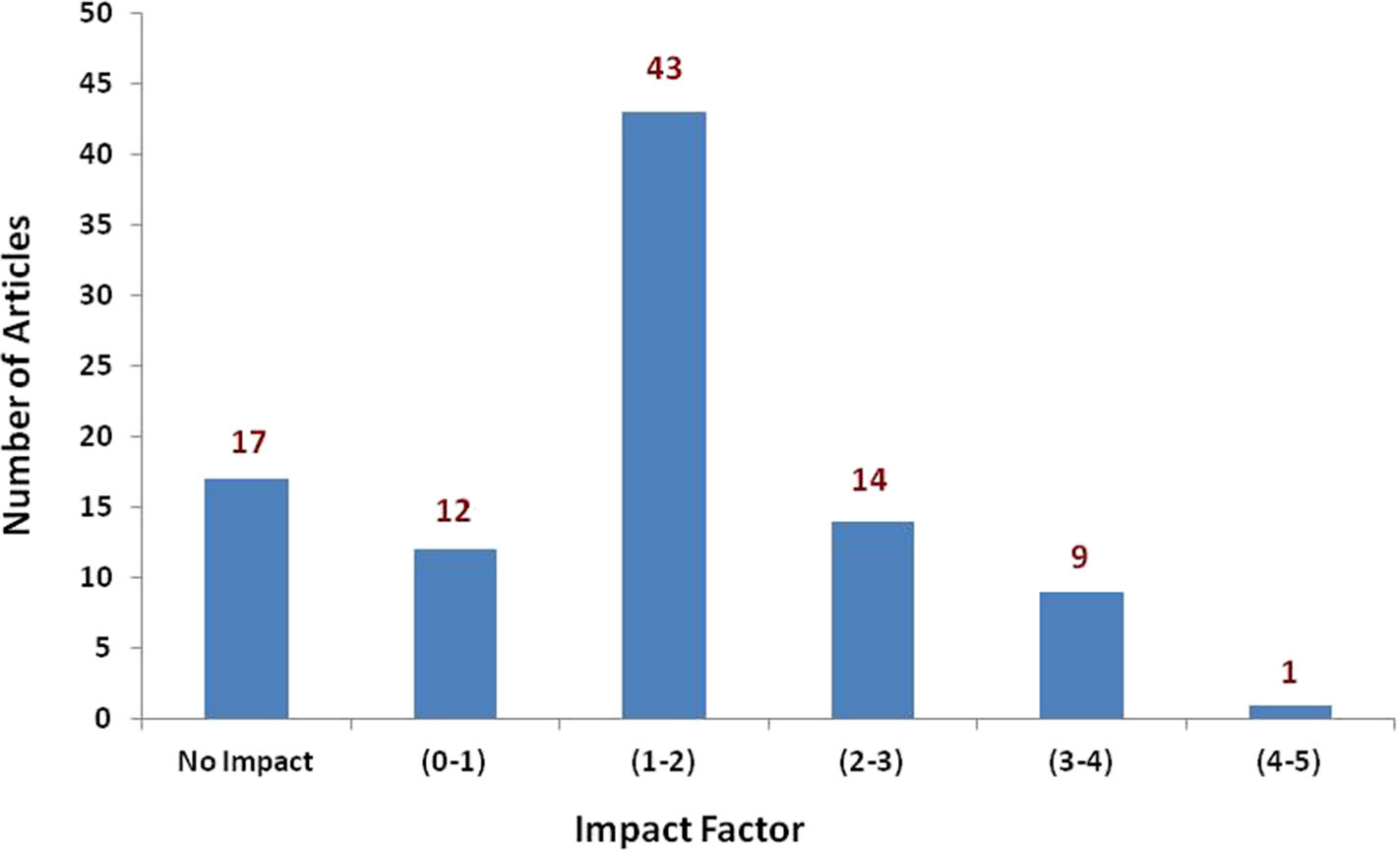
The impact factors of the urology publications in the UAE.

As demonstrated in Table 1, more than two thirds of the articles were original articles whereas case reports constituted one quarter of the published urological articles. Sixty four articles (67%) represented clinical whereas 32 articles (33%) represented laboratory-based research. As demonstrated in Table 2, out of all subspecialties, urinary obstruction and urolithiasis represented the most common field of research. None of the articles represented a proper epidemiological research.As shown in Figure 2, almost half of the articles were published in journals with impact factors between one and two whereas only one articles was published in a journal with an impact factor of more than four.There was a trend for the research activities, in general, and in any particular field, to be sporadic and to last for a relatively short period of time due to the fact that such activities were usually driven by certain research-active individuals; the departure of these individuals caused a cessation of the research activity. This was demonstrated by several examples such as the transient increase in the research activities in Dubai Hospital in the period between 2006 and 2007 and in the central hospital between 1985 and 1992 as shown in Figure 1.

**Table 1:** Type of urology publications from the UAE

**Type of publication**	**No (%)**
Original research articles	68 (71%)
Case reports	24 (25%)
Review articles	4 (4%)

**Table 2:** The various fields of the urology publications from the UAE

**Field**	**No (%)**
Urinary obstruction and urolithiasis	19 (20%)
Female urology	14 (15%)
Congenital abnormalities	12 (13%)
Andrology	11 (11%)
Voiding dysfunction	10 (10%)
Uro-oncology	8 (8%)
Inflammation and infection	6 (6%)
Renal ischemia and transplantation	6 (6%)
Genitourinary trauma	5 (5%)
Others	5 (5%)

## Discussion

This study has shown that there was an overall increase in the number of publications in Urology in this country overtime, which paralleled the increase in the population size and number of urologists in the country. However, there appears to be a lack of urology research groups as demonstrated by the cessation of research activity by the departure of certain research-active individuals. Such research groups would not only improve the local research standards, but would also ensure the continuity of research activity even if certain individuals need to leave the country due to personal reasons which is not uncommon in the Gulf countries and other developing nations and could be due to immigration in a trail to find better career opportunities.

One of remarkable observations was the lack of proper epidemiological studies which is required to establish the prevalence of various diseases and their impact on the national population. This is in contrast to other subspecialties such traumatology where the majority of studies were of epidemiological nature [5]. The reason for this discrepancy is difficult to ascertain but might be due the inherent differences between these two specialities. Alternatively, it could be due to coincident existence of epidemiology-oriented individuals in the trauma compared to urology service in the country. Should this be the case, this also supports the fact that the research is driven by personal interests and expertise and not by the national needs.

The current study has demonstrated that the majority of urology publications from this country came from the main academic institute (UAE University). This is probably expected due to the relatively research intensive nature of this institute and the availability of well-equipped laboratories. This trend appears to be similar to the one observed when the overall medical research publications from the Gulf Corporation Council (GCC) countries were analysed [6]. Therefore, individuals from outside academic institutes who are interested in research activities might do better by collaborating with individuals from these institutes. This fact might also put some responsibility on the academic institutes in the region to extend their collaborations to non-academic institutes to enhance the research activities and standards in the area. The fact that the majority of the studies in the country had come from the main academic institute which has proper research facilities, might also explain the relatively good impact factor of the journals where these articles were published.

From the current study, it appears that there was disproportionately higher percentage of case reports compared to original research articles. The most likely reason for this is the lack of adequate experience to perform proper research. Alternatively, it might be due to the lack of adequate number of cases in this relatively small country to enable the urologist to undergo clinical trials especially in view of the lack of centralization of clinical services and the scarcity of tertiary referral centres. Certainly, centralising the clinical services would not only improve the clinical outcome but might also improve the local research productivity.

The distribution of the publications’ research fields in this study appears to mirror the distribution of urological diseases in the local set-up. For instance, there is a strong correlation between stone formation and hot climate [7,8] which is one of the characteristics of this part of the world. This is reflected by a high proportion of publications in this filed. Similarly, and due to the pro-natal nature of the community, there appears to be a relatively high proportion of publications in the field of andrology including infertility. Surprisingly, however, there was a small number of publications dealing with genitourinary trauma despite the fact that trauma, in general, is considered as a major cause of death in this country [9]. This is probably due to the fact that genitourinary organs are exceptionally rarely affected in trauma as demonstrated by some of the local studies [10,11].

Although it might have been expected to find a small number of laboratory-based research activities in this relatively newly-developed country, it appears that one third of the publications have reflected laboratory-based research. The majority of this type of research originated from the main university institute probably reflecting the existence of relatively well-equipped research laboratories in that particular institute.

Finally, some of the trends observed in this study appear to be similar to the existing trends in other clinical sub-specialties in other countries in the region [12-15]. This is probably due to the similarity in the disease distribution and physicians training backgrounds. Such trends need to be recognised by policy-making institutes to improve the research standards and to maximise the national benefit of the research projects.

## Conclusion

In conclusion, there has been a recent increase in the number of published articles in Urology. Generally, the fields of research in these articles appear to reflect the distribution of urological diseases in the local set-up. However, there is a lack of proper epidemiological studies to determine the incidence of urological diseases and their impact on the local population and therefore, there is a need for a strategic plan to support research in this area. In addition, there is a lack of research groups and hence a lack of continuity of research in any particular field as the research is usually driven by specific research-oriented individuals the departure of who results in a cease in the research activities.

## Competing interests

The author declares that he has no competing interests to disclose.
